# Regional Lung Perfusion as Determined by Electrical Impedance Tomography in Comparison With Electron Beam CT Imaging

**DOI:** 10.1109/TMI.2002.800585

**Published:** 2019-09-13

**Authors:** Inéz Frerichs, José Hinz, Peter Herrmann, Gerald Weisser, Günter Hahn, Michael Quintel, Gerhard Hellige

**Affiliations:** 1 Department of Anesthesiological ResearchEmergency and Intensive Care MedicineCenter of Anesthesiology TL 195; 2 Robert-Koch-Strasse 40University of Göttingen D-37075GöttingenGermany; 3 Department of Anesthesiological Research Center of Anesthesiology Emergency and Intensive Care MedicineUniversity of Göttingen D-37075GöttingenGermany; 4 Institute of Anesthesiology and Operative Intensive CareUniversity Hospital Theodor-Kutzer-Ufer68167MannheimGermany; 5 Institute of Clinical RadiologyUniversity Hospital Theodor-Kutzer-Ufer68167MannheimGermany

**Keywords:** EIT, functional imaging, perfusion defect, pulmonary embolism, pulmonary flow, thoracic impedance

## Abstract

The aim of the experiments was to check the feasibility of pulmonary perfusion imaging by functional electrical impedance tomography (EIT) and to compare the EIT findings with electron beam computed tomography (EBCT) scans. In three pigs, a Swan-Ganz catheter was positioned in a pulmonary artery branch and hypertonic saline solution or a radiographic contrast agent were administered as boli through the distal or proximal openings of the catheter. During the administration through the proximal opening, the balloon at the tip of the catheter was either deflated or inflated. The latter case represented a perfusion defect. The series of EIT scans of the momentary distribution of electrical impedance within the chest were obtained during each saline bolus administration at a rate of 13/s. EBCT scans were acquired at a rate of 3.3/s during bolus administrations of the radiopaque contrast material under the same steady-state conditions. The EIT data were used to generate local time-impedance curves and functional EIT images showing the perfusion of a small lung region, both lungs with a perfusion defect and complete both lungs during bolus administration through the distal and proximal catheter opening with an inflated or deflated balloon, respectively. The results indicate that EIT imaging of lung perfusion is feasible when an electrical impedance contrast agent is used.

## I. Introduction

The passage of blood through the pulmonary blood vessels as well as the periodic changes of the blood volume and the distension of the vessel walls associated with heart action are, besides the ventilation-related air volume changes, one of the major causes of the *in vivo* variation of electrical properties of lung tissue with time [1]. The electrical properties of biological tissues can be determined by the technique of electrical impedance tomography (EIT) with a very good time resolution. EIT generates two-dimensional images of the distribution of electrical impedance within a studied body section at rates approaching a few tens of EIT images/s. Until now, the imaging of lung ventilation has dominated the chest applications of EIT [2], nevertheless, a few examinations aiming at imaging pulmonary perfusion have been performed as well [1], [3]–[6].

The most probable future use of EIT in imaging lung perfusion is the detection of perfusion defects in pulmonary embolism [7]. Acute lung embolism is a life-threatening disease which requires prompt initiation of, e.g., anticoagulant and thrombolytic therapy. However, in a large group of patients (e.g., the post-operative ones), this therapy may be associated with severe side effects because of increased bleeding risk. So as not to unnecessarily post-pone the onset of the possibly life-saving therapy, a rapid and certain diagnosis of pulmonary embolism using a method with high sensitivity and specificity would be desirable.

As far as the imaging techniques are concerned, pulmonary angiography and perfusion scintigraphy are the methods of choice. In the recent years, the use of computed tomography (CT) and magnetic resonance tomography (MRT) is also being recommended [8] [9] [10]. However, none of these methods allows the early bedside diagnosis of pulmonary embolism and the continuous monitoring of the therapy effect. Moreover, these techniques are not universally available, expensive and their use is associated with complications and side effects (e.g., adverse reactions to the radiographic contrast material, increased radiation load).

The modern EIT devices are noninvasive, small, and inexpensive. They allow measurements to be performed without any known hazard directly at the bedside. We have checked the feasibility of examining the normal and defective lung perfusion by functional EIT in animal experiments using a new methodological approach. Our method for the visualization of regional blood flow in the lungs by EIT is based on the indicator dilution technique and it uses an electrically conductive fluid as a contrast agent. The EIT findings are compared with electron beam computed tomography (EBCT) scans.

## II. Methods

Measurements were performed on three anesthetized supine pigs (20-kg mean body weight). The experiments were approved by the university and government ethical committees and adhered to the guidelines on animal experimentation. The animals were initially sedated by azaperon (0.25-ml/kg body weight). Anesthesia was performed by a continuous intravenous administration of ketamine (200 mg/hr) and propofol (100 mg/hr). Muscle paralysis was achieved by vecuronium bromide (0.1-mg/kg body weight). The animals were tracheo-tomized, intubated and mechanically ventilated at a rate of 12 breaths/min, a tidal volume of 240 ml, an inspiration/expiration ratio of 1/1.3, and positive end-expiratory pressure of 5-cm $\hbox{H}_{2}\hbox{O}$. The left carotid artery and the right external jugular vein were cannulated. A Swan-Ganz catheter was introduced into a branch of the right or the left pulmonary artery and its position checked by EBCT.

### A. EIT Measurements

The EIT measurements were performed with the Goettingen Goe-MF 1 EIT system. This is a 16-electrode multiplexed EIT system whose characteristics and performance are described in [11]. This device has previously been used in a study on healthy humans during parabolic flights [12]. In the present experiments, 16 X-ray transparent electrocardiogram (ECG) electrodes (Blue Sensor BR-50-K, Medicotest A/S, Ølstykke, Denmark) were placed around the boundary of a transverse thoracic plane lying approximately 1 cm below the tip of the Swan-Ganz catheter. (The proper location of the electrodes was established on the basis of a previously acquired planar EBCT chest image.) The array of surface electrodes was needed by the EIT technique for the rotating injection of small alternating electrical currents ($\hbox{5}~mA _{\rm p-p}$, 50 kHz) and the measurement of resulting potential differences. The injection of the excitation currents was consecutively performed through all 16 adjacent electrode pairs. Each current application was followed by the voltage measurements between all adjacent electrodes except the driving ones. One complete cycle of current injections and voltage measurements was completed within 77 ms. A total of 1000 consecutive measuring cycles were performed during an individual EIT measurement, i.e., the duration of one measurement was 77 s.

The momentary distributions of electrical impedance within the chest were calculated from the EIT data collected during each measuring cycle with an image matrix of 32×32 pixels using a back-projection algorithm. (In fact, the reconstruction algorithm used provided the relative values of local impedance change with respect to a reference state of impedance distribution [13].) Each EIT measurement gave a sequence of 1000 consecutive images of electrical impedance distributions at a rate of 13/s.

### B. EBCT Measurements

The EBCT measurements were performed with the EBCT scanner C-150XP (Imatron, San Fransisco, CA) using the so-called multislice mode of scanning. This scanning protocol did not require the movement of the animal through the scanner. During an individual EBCT measurement, a total of 40 scans in four chest slices (individual width: 7 mm) each were acquired at a rate of 3.3 scans/s. Thus, the whole scan period lasted 12 s which is the maximum duration of data acquisition achievable with the scanning parameters chosen. The scan mode allowed four transverse scans with 256×256 pixels to be generated at each time point. The exposure time was 50 ms/slice. The EBCT data spanned 3.8 cm of the chest/time point.

### C. Contrast Agents

Contrast agents were administered as boli through the distal or the proximal openings of the Swan-Ganz catheter. An electrically conductive agent (5.85% saline solution) was injected as an indicator during the EIT measurements, whereas radiographic contrast material (Ultravist, Schering, Berlin, Germany) was used during the EBCT measurements. The volume of the boli injected was 15 and 20 ml during administration through the distal and proximal catheter opening, respectively. The boli of the hyper-tonic saline solution were hand injected, the radiopaque contrast agent was administered by an automated injector at a rate of 5 ml/s.

### D. Protocol

EIT and EBCT measurements were always performed one after each other under the same steady-state experimental conditions.

1 During the first measurement, the data were acquired in the course of undisturbed mechanical ventilation.
  During the subsequent measurements, described below, the mechanical ventilation was temporarily interrupted and EIT as well as EBCT scanning was performed at end-expiration.
2 A bolus of the contrast agent was administered through the distal opening of the Swan-Ganz catheter.
3**Fig. 1.**
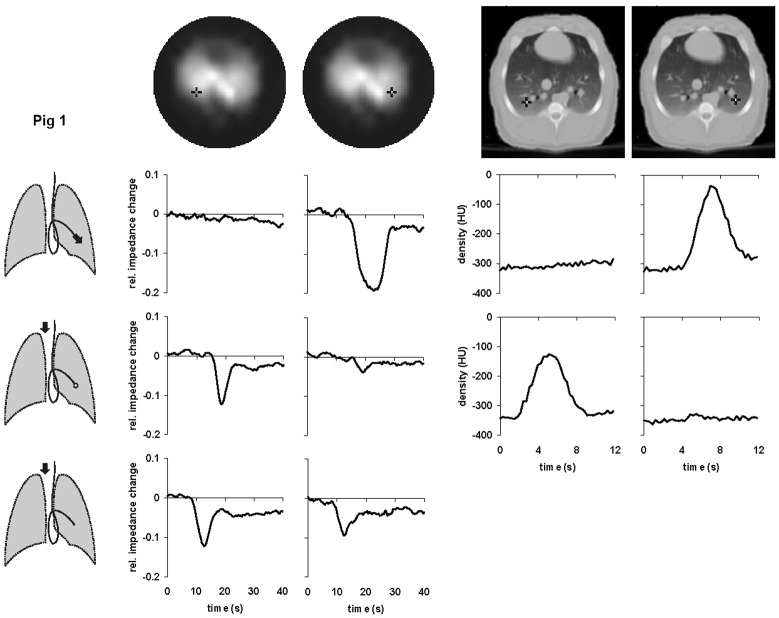
Local time-impedance (six left-hand diagrams) and time-density curves (four right-hand diagrams) in pig 1 during bolus administration of the hypertonic saline solution and the radiographic contrast material, respectively. The black crosses in the dorsal regions of the right and left lungs in the functional EIT images of lung ventilation and in the EBCT images (top) indicate the pixel locations at which the data were obtained. Three drawings of the lungs show schematically the position of the Swan-Ganz catheter in a branch of the left pulmonary artery and the bolus administration sites (black arrows). The bolus was administered either through the distal (upper drawing) or the proximal opening of the catheter with the balloon at the tip of the catheter being inflated (middle drawing) or deflated (lower drawing). The EBCT measurements during the central venous bolus administration with deflated balloon were not performed in this animal.

**Fig. 2.**
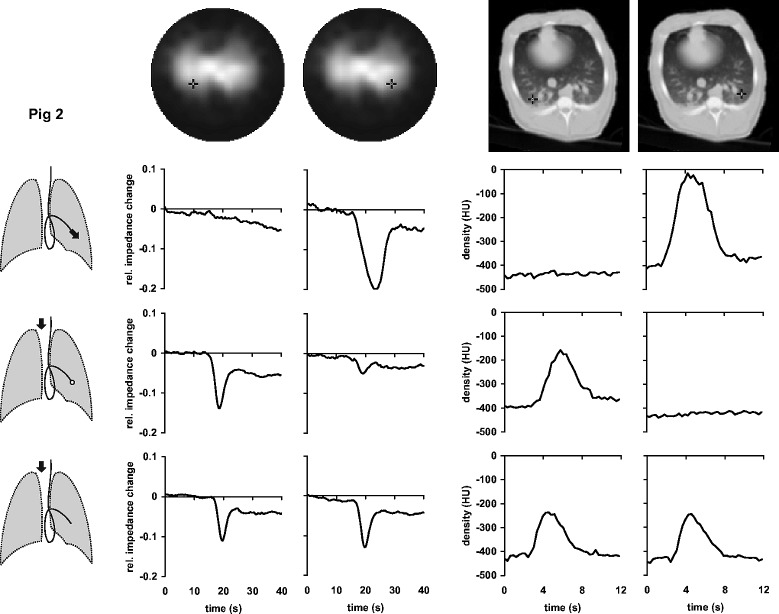
Local time-impedance (six left-hand diagrams) and time-density curves (six right-hand diagrams) in pig 2 during bolus administration of the hypertonic saline solution and the radiographic contrast material, respectively. The black crosses in the dorsal regions of the right and left lungs in the functional EIT images of lung ventilation and in the EBCT images (top) indicate the pixel locations at which the data were obtained. Three drawings of the lungs show schematically the position of the Swan-Ganz catheter in a branch of the left pulmonary artery and the bolus administration sites (black arrows). The bolus was administered either through the distal (upper drawing) or the proximal opening of the catheter with the balloon at the tip of the catheter being inflated (middle drawing) or deflated (lower drawing).

**Fig. 3.**
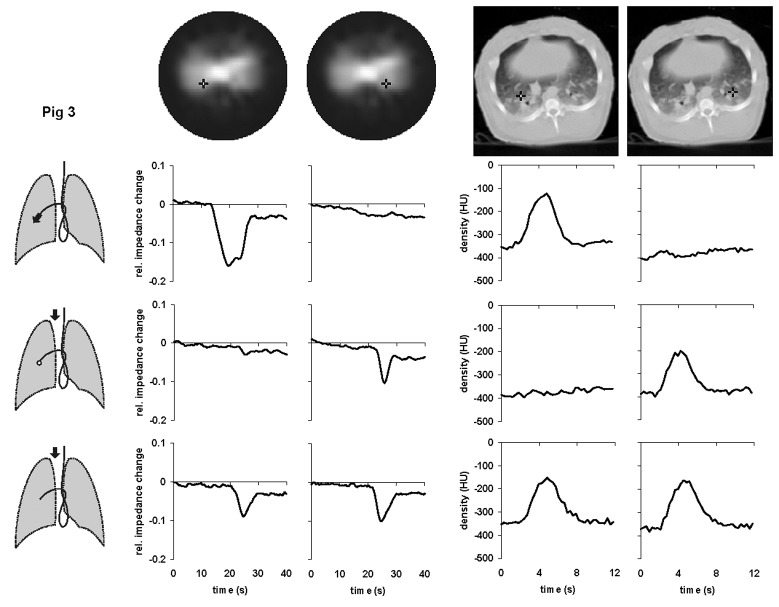
Local time-impedance (six left-hand diagrams) and time-density curves (six right-hand diagrams) in pig 3 during bolus administration of the hypertonic saline solution and the radiographic contrast material, respectively. The black crosses in the dorsal regions of the right and left lungs in the functional EIT images of lung ventilation and in the EBCT images (top) indicate the pixel locations at which the data were obtained. Three drawings of the lungs show schematically the position of the Swan-Ganz catheter in a branch of the right pulmonary artery and the bolus administration sites (black arrows). The bolus was administered either through the distal (upper drawing) or the proximal opening of the catheter with the balloon at the tip of the catheter being inflated (middle drawing) or deflated (*lower drawing*).

**Fig. 4.**
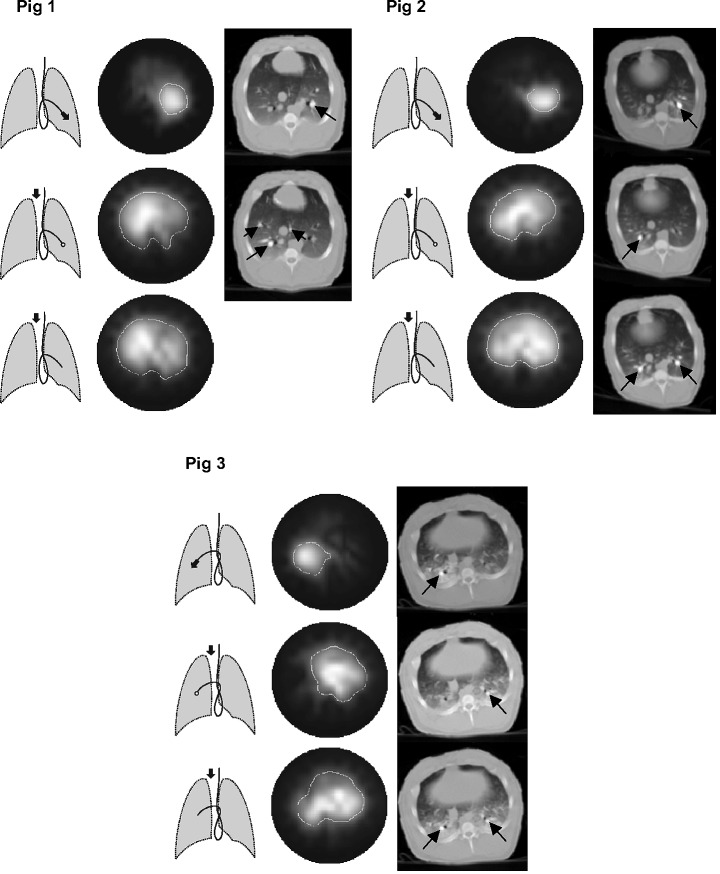
Functional EIT images showing the regional blood flow in three pigs during administration of the hyper-tonic saline solution at different administration sites (see the schematic drawings of lungs next to the EIT images). The EIT images show in light tones those regions in the thoracic cross section where a fall of electrical impedance was observed during administration of a saline bolus. The EBCT scans show the respective chest cross sections at the moment of appearance of the contrast enhancement in the pulmonary arterial system (black arrows) during bolus administration at the sites indicated by the drawings of lungs and corresponding with the EIT measurements.

The balloon at the tip of the catheter was inflated causing a transient interruption of the blood flow through the occluded artery. Immediately afterwards, a bolus was administered through the proximal opening of the catheter.
4 A bolus was injected through the proximal opening of the catheter with the balloon being deflated.

In all measurements, the data acquisition was initiated shortly before the injection of a bolus and spanned the pulmonary arrival and outflow of the agent.

### E. Data Evaluation

A functional EIT image of regional lung ventilation was generated from the sequence of distributions of electrical impedance within the chest cross section acquired during mechanical ventilation using an evaluation approach based on the calculation of the local end-inspiratory-to-end-expiratory impedance changes [12], [13]. This type of functional EIT image shows the ventilated lung regions in light tones [see Figs. 1–3(top)].

The EIT data obtained in the subsequent measurements during boli administrations were used to generate local time-impedance dilution curves and a new type of a functional EIT image visualizing the lung regions with a significant decline in electrical impedance on the bolus arrival. This image was created by subtracting the EIT data obtained prior to bolus injection from those obtained at the full bolus arrival. The regions manifesting a large decrease in impedance were depicted in light tones (see Fig. 4).

Local time-density curves were generated from each EBCT measurement using a procedure similar to [15]. The EBCT scans showing the arrival of the radiographic material in the pulmonary arterial system and the contrast enhancement in the lung tissue were identified in the sequences of acquired scans and used as a visual reference for the EIT measurements. They showed the exact location of those lung regions into which the indicator was carried by the blood stream.

## III. Results

A total of three animals were studied by EIT during different steady-state experimental conditions. Normal lung circulation and simulated occlusion of a pulmonary artery branch were followed during breath-holding utilizing the principle of the indicator dilution technique. Additional measurements were performed during undisturbed mechanical ventilation. The EIT findings were compared with the reference EBCT measurements.

Figs. 1–3 show the local time-impedance and time-density curves obtained in the studied animals during three different types of bolus administration of the nonradiographic and radiographic contrast agents, respectively. (For better comparison with the EBCT curves obtained during a brief data acquisition period, only the first 40 s of the EIT measurements are shown.) The curves shown were generated from the EIT and EBCT data at two pixel locations in the right and left lung. One pixel was situated in a lung region supplied by the vessel in which the catheter tip was located, the other one was in the contralateral lung at the same vertical height as the first one. In all animals, an ipsilateral fall of electrical impedance was observed during injection of the saline solution through the distal opening of the catheter whereas no such impedance change was detected in the contralateral lung. During administration through the proximal opening with the catheter balloon being inflated, the bolus did not arrive in the lung periphery supplied by the blocked vessel and the fall in regional electrical impedance was found on the opposite side than during the first type of bolus administration. When the balloon was deflated and bolus injected through the proximal opening, regional electrical impedance decreased in both lungs. The time-density curves obtained from the EBCT data exhibited essentially the same characteristics as the EIT findings.

The functional EIT images generated from the data obtained during bolus administrations of the conductive contrast agent at different injection sites are shown in Fig. 4. For comparison, the EBCT scans taken at the moment of arrival of the radiographic contrast material in the pulmonary arterial system during administration at injection sites identical with the EIT measure-ments are presented in Fig. 4 as well. During administration through the distal catheter opening, only the blood flow through the lung region lying peripherally to the injection site was visualized. When the catheter balloon was inflated, the administration of the bolus through the proximal opening resulted in an image showing the blood flow through the lungs and the heart with a perfusion deficit in the blocked lung region. Bolus administration through the proximal catheter opening with a deflated balloon showed the undisturbed blood flow in the whole chest cross section.

## IV. Discussion

EIT imaging of pulmonary perfusion is a methodologically demanding task. Not only are the electrical impedance changes associated with lung perfusion small but they are also masked and modulated by the much larger changes related to lung ventilation. In most of the EIT studies aimed at assessing lung perfusion, the ECG-triggered data acquisition was used for the period of 100–200 cardiac cycles and the effect of ventilation was minimized by averaging [1], [3], [6]. The impedance changes occurring synchronously with the heart rate, usually termed as “perfusion-related impedance changes,” can also be separated from the ventilation-related ones by digital filtering [4], [5]. The other approach used to minimize the ventilation component of the impedance signal, used in the present experiments as well, is to acquire the EIT data during breath-holding.

Nonetheless, even when the effect of lung volume changes on the measured thoracic EIT data is attenuated by the mentioned procedures, the perfusion-related variation of electrical impedance remains small and hard to detect. Moreover, the complex origin of the acquired impedance data does not allow to separate the true perfusion from other components contributing to the cardiac synchronous impedance changes. The use of an intravascular contrast material, which in the case of EIT perfusion imaging means an agent with either higher or lower conductivity than blood, could provide a large impedance signal related directly to pulmonary blood flow. Previous *in vitro* experiments have shown that EIT can follow the passage of an isotonic saline solution bolus through a tube placed in a tank filled with a solution with conductivity comparable to blood [16]. It was even demonstrated that the saline passage can be detected in the upper arm veins and in the cardiac region in one subject. Based on this approach, we have used the hypertonic saline solution applied as a contrast agent to follow local pulmonary perfusion in our experiments.

We have administered a bolus of the hypertonic saline solution at different anatomical sites including the pulmonary arterial and central venous systems by means of a multihole catheter. Our experimental procedure enabled us to study the normal lung perfusion and to simulate a perfusion defect. We were not only able to visualize the blood flow through both lungs and a selected small lung region but also to detect a region of the blood flow deficit. The EBCT scans allowed us to exactly locate the passage of the administered EIT bolus contrast material in the chest cross section by using a radiopaque agent under identical experimental conditions. The acquisition of sequences of EIT data during the arrival and outflow of the bolus into and from lungs enabled us to generate regional time-impedance curves.

The obtained results and the comparison with an established imaging technique clearly show the potential of EIT for a new meaningful clinical application. The experiments indicate that EIT assessment of pulmonary perfusion and the detection of perfusion defects is possible. New functional EIT images visualizing the regional pulmonary blood flow were generated. In the present experiments, the peak impedance change on the bolus arrival in lungs was used for the simple identification of those lung regions where the indicator was carried to by the blood stream. However, the ability of EIT to follow the transit of a bolus through the pulmonary vascular system in terms of local time-impedance curves indicates that quantitative analysis of EIT data based on the indicator dilution principle may be attempted in the future. The calculation of the area under the time-impedance dilution curve which is inversely proportional with the flux as well as of other specific curve parameters like appearance time, appearance-to-peak time and mean transit time should be possible. Because EIT is able to produce images of lung ventilation [2], the present findings also indicate that multifunctional imaging of the lung function by EIT comparable with the isotope ventilation/perfusion scans may be aspired in the future. Functional EIT images have a similar resolution than the scintigraphic scans, however, their advantage is that they can be obtained at the bedside at low cost and without the use of radioactive tracers. At present it is too early to define the exact clinical perspective of EIT in this new indication with certainty.

We believe that further basic studies will prove that EIT can be used as a method for analyzing lung perfusion. Studies on larger groups of experimental animals designed to establish the ability of functional EIT to quantitatively assess the local blood flow in comparison with reference techniques are necessary.
